# A Comparative Study of Histotripsy Parameters for the Treatment of Fibrotic ex-vivo Human Benign Prostatic Hyperplasia Tissue

**DOI:** 10.21203/rs.3.rs-4549536/v1

**Published:** 2024-07-02

**Authors:** Yashwanth Nanda Kumar, Zorawar Singh, Yak-Nam Wang, Diboro Kanabolo, Lucas Chen, Matthew Bruce, Eli Vlaisavljevich, Lawrence True, Adam D. Maxwell, George R. Schade

**Affiliations:** University of Washington; University of Washington; University of Washington; University of Washington; University of Washington; University of Washington; Virginia Polytechnic Institute and State University; University of Washington; University of Washington; University of Washington

**Keywords:** Fibrotic tissue, Benign prostate hyperplasia, Cavitation cloud histotripsy, Boling history, Shear wave elastography, Entropy-based lesion quantification

## Abstract

Histotripsy is a noninvasive focused ultrasound therapy that mechanically fractionates tissue to create well-defined lesions. In a previous clinical pilot trial to treat benign prostatic hyperplasia (BPH), histotripsy did not result in consistent objective improvements in symptoms, potentially because of the fibrotic and mechanically tough nature of this tissue. In this study, we aimed to identify the dosage required to homogenize BPH tissue by different histotripsy modalities, including boiling histotripsy (BH) and cavitation histotripsy (CH). A method for histotripsy lesion quantification via entropy (HLQE) analysis was developed and utilized to quantify lesion area of the respective treatments. These data were correlated to changes in mechanical stiffness measured by ultrasound shear-wave elastography before and after treatment with each parameter set and dose. Time points corresponding to histologically observed complete lesions were qualitatively evaluated and quantitatively measured. For the BH treatment, complete lesions occurred with >=30s treatment time, with a corresponding maximum reduction in stiffness of −90.9±7.2(s.d.)%. High pulse repetition frequency (PRF) CH achieved a similar reduction to that of BH at 288s (−91.6±6.0(s.d.)%), and low-PRF CH achieved a (−82.1±5.1(s.d.)%) reduction in stiffness at dose >=144s. Receiver operating characteristic curve analysis showed that a >~75% reduction in stiffness positively correlated with complete lesions observed histologically, and can provide an alternative metric to track treatment progression.

## Introduction

Benign prostate hyperplasia (BPH) is a noncancerous enlargement of the prostate gland, wherein an imbalance in cell proliferation and cell death leads to an increase in the epithelial and stromal cell count in the periurethral area of the prostate. It is the only solid organ to grow throughout a man’s life, and thus, the incidence of BPH increases dramatically with age, particularly in individuals over 60 years of age. BPH is among the top 5 noncancer-related disorders for men^1^. While BPH in many cases may be asymptomatic and not disruptive, it is one of the leading contributors to lower urinary tract symptoms (LUTS), thus affecting quality of life and increasing the chances of associated medical morbidities. Some LUTS include increased frequency, urgency to urinate, nocturia, painful urination, lack of voluntary control, longer voiding, urinary retention and weak flow of urine^2^. Many live with BPH without treatment^3^, but doing so can lead to complications such as urinary tract infections, hematuria, urinary retention, and urinary stasis, leading to stone formation and contributing to kidney failure^4,5^.

Treatment options for BPH include drug-based interventions such as alpha blockers that relax the smooth muscles associated with the urinary bladder and 5-alpha reductase inhibitors that reduce prostate volume by inhibiting synthesis of the more potent dihydrotestosterone^6^ to facilitate urinary voiding. However, these interventions are not always effective due to a variety of factors. One study revealed that approximately 30% of men stop medication within 2 years^7^. Drug-associated side effects such as dizziness, fatigue, and asthenia discourage medication use when the prostate size is too large to be effective^8,9^. An alternative treatment involves surgical intervention, such as transurethral resection of the prostate (TURP), which is considered the gold standard of therapy^10^, monopolar and bipolar laser-based vaporization, resection and enucleation of the prostate, and simple prostatectomy. However, surgical interventions can lead to postsurgical complications, particularly in men with comorbidities^11^. Many of these patients would therefore benefit from less invasive options.

Histotripsy is a noninvasive, nonionizing focused ultrasound therapy that mechanically fractionates tissue and creates precise lesions by generating bubble clouds in the tissue. Histotripsy can be performed through cavitation or boiling to generate bubbles. Cavitation histotripsy (CH) involves the application of microsecond-length pulses of high pressure amplitude at a low duty cycle to produce dense bubble clouds via intrinsic nucleation or shock scattering ^12,13^. The repeated expansion and contraction of the bubbles impart strain on the surrounding tissue, thus disintegrating it to a subcellular level, effectively homogenizing tissue, which can then be passed through the colon as feces or reabsorbed by the body. Boiling histotripsy (BH) uses longer millisecond-length pulses to rapidly generate millimeter-sized boiling bubbles and achieve a similar outcome. High-amplitude shock waves produce heat that reduces the intrinsic threshold required for bubble nucleation, and the subsequent interaction of the shock fronts with the resulting vapor cavity causes tissue disintegration^14,15^. Both CH and BH modalities have been demonstrated in preclinical studies to treat a variety of tissues, such as porcine liver, kidney, hematomas, abscesses, and prostate ^14,16–21^.

A clinical system was developed, and a trial was conducted to treat BPH with CH modality. Preclinical optimization studies demonstrated good ablation and safety using high-frequency platelet (PRF) (500 Hz) short pulse (3 cycles) parameters^22^ and eventually led to a clinical trial of this treatment^23^. This clinical trial demonstrated the safety of the treatment; however, it did not show objective quantitative improvements in symptoms. One potential reason for the lack of improvement was that the treatment parameters optimized in a canine model were not effective for treating human BPH. In human BPH, the fibromuscular stroma is often the dominant tissue, which makes it tougher and more resilient to histotripsy treatment^24,25^. Additionally, the smaller acoustic window for ultrasound to propagate through and thicker overlying tissue in humans than in dogs may have contributed to a lack of ultrasonic power.

Both CH and BH can spare fibrous tissue^26^, but other studies have demonstrated complete disintegration of fibrous tissues, such as Peyronie’s plaque^27^, ureteroceles^28^, cholangiocarcinoma^29^, uterine fibroids^30^ etc., requiring a higher dose per point. A recent pilot study has also shown the ability of BH to ablate the *ex vivo* human BPH prostate ^31^. The inconsistency between these studies indicates that a gap in knowledge exists in choosing the right treatment parameters to produce safe and effective ablation of these different tissues. In this study, we aimed to compare three sets of treatment parameters to determine lesion progression as a function of dosage and evaluate the outcomes histologically and by stiffness measurements from shear wave elastography (SWE). For histological analysis, we developed a hybrid entropy-based texture analysis algorithm combined with Otsu’s thresholding to automatically segment and identify homogenate lesion from surrounding tissue which also took into account the stain inconsistencies.

## Results

In this study, we explored different parameter sets across cavitation CH and BH as described in Table 1 at various doses to treat ex-vivo human prostate samples (N = 23) obtained from patients undergoing simple prostatectomy procedures for BPH using an IRB exempt tissue procurement protocol (NWBiotrust, Seattle, WA). Two different transducers were used to deliver the respective therapies with image guidance provided by in-line off the shelf commercial imagers. Shear wave elastography (SWE) was used to measure tissue stiffness before and after treatment to assess the treatment outcome and the tissue was later fixed and sent to histology for Masson’s trichrome staining. Additionally, a novel processing algorithm was developed, named the Histotripsy Lesion Quantification by Entropy analysis (HLQE) to quantify the lesion as a direct measure of the treatment outcome.

**Figure 1:**
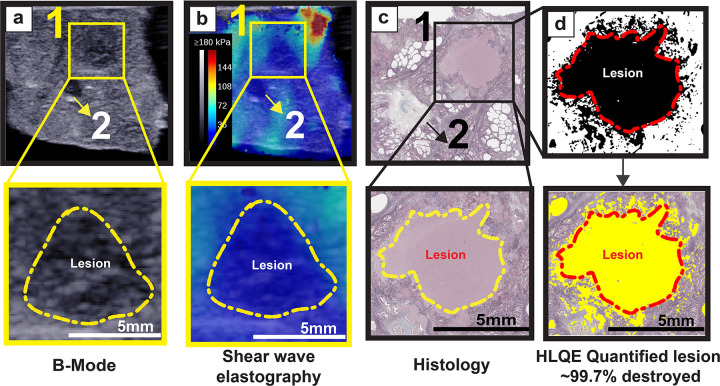
Lesion visualization modalities: **(a)-(c)** show the lesion appearance across B mode, shear wave elastography and histology. Identified here are two treatment areas with grid 1 receiving BH at 60s and grid 2 receiving BH at 30s. Both the treatment grids can be visibly identified (by the yellow/red outline) and are distinguishable on all the modalities. **(d)** binary map of the lesion identified by the HLQE algorithm and superimposed on the histology slice.

During treatment, bubble clouds appear as hyperechoic regions on the B-mode, with no significant change in appearance of tissue at lower treatment dose. Figure 1(a),(b) summarize the appearance of a complete lesion generated by BH across the B-mode and SWE. In B-mode, the fully developed lesion appears as a hypoechoic zone, which is also reflected in the shear wave image, where there is a clear contrast in the colormap. Shown in Fig. 1(c) a representative image of Masson’s trichrome stained section with lesion site identified. Figure 1(d) shows the performance of the HLQE algorithm in identifying the lesion and calculating the % destroyed tissue. The top row of (d) shows the binary image produced from thresholding the entropy calculated across the lesion grid, with the bottom panel showing the superimposed binary image onto the actual histology slice, with red outline representing the lesion area. There is a slight difference in the lesion outlines between the imaging modalities and histology, partially due to shrinkage of tissue during formalin fixation and artefacts during processing for staining.

### Histological evaluation.

**Figure 2:**
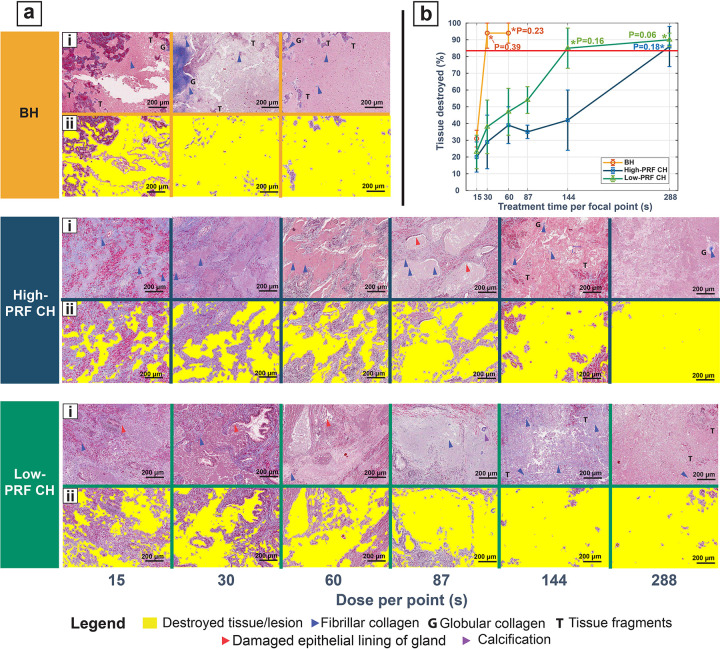
Appearance of histotripsy treated prostate by histology. **(a)(i)** shows the Masson’s trichrome stained sections of EVHP samples subjected tovarious exposure parameters and treatment times. The blue arrows indicate the presence of intact fibrillar collagen, with **“G”** indicating globular collagen, **“T”** pointing to tissue fragments. Red arrows point to the damaged epitheliallining of the glands. **a(ii)** is the superimposed lesion image detected by the HLQE algorithm. **(b)** Average percentage of tissue destroyed across different doses (time per focus) based on areal analysis of the histological sections using the HLQE algorithm. *p-values for the one sample t-test, where the population mean is statistically indistinguishable from a mean value= 100%. The red line indicates the minimum average tissue destroyed (%) set as the minimum threshold required to classify as a complete lesion.

Both qualitative and quantitative metrics were evaluated across all treatment parameter sets and dose. Figure 2(a) presents a detailed picture of the progression of tissue damage for each parameter set with an increase in dose. **Panel (i)** is the Masson’s trichrome stained slide with important treatment-related features. **Panel (ii)** shows the lesion map detected by the HLQE algorithm superimposed on the histology section.

### Qualitative analysis.

With increasing dose, small, isolated regions of damage progress to contiguous larger lesions at higher doses. A fully homogenized lesion was observed at 288s for high-PRF CH, ≥144s for the low-PRF CH and at ≥30s for the BH. All histotripsy parameter sets produced lesions with high fidelity to cover the entire intended area of treatment plan.

- *Lesion border distinction*: In both BH and CH treatments, a clearly distinguished lesion was found if the treatment was entirely within a glandular region of the tissue. Treatments in fibromuscular bands showed a lesion surrounded by a less distinct border with multifocal areas of homogenization and frayed collagen and muscle fibers. All BH treatments showed presence of frayed collagen within the lesion (blue arrows) at all doses, however in CH treatments they tend to appear less frequently at higher doses .
- *Globular collagen with increasing dose and duty cycle*: Globular collagen was observed in tissue treated with BH (1% duty cycle) at a dose of 30 s (Fig. 2 (a) BH 30 and 60s. Some amount of globular collagen was also observed in the High-PRF CH treatments (duty cycle-0.21%) at 144s and more prominently at 288 s (Fig. 2 (a) High-PRF CH (ii) 144 s and 288 s). However, in the low-PRF CH treatments, globular collagen was not observed.
- *Tissue fragments*: In BH treatments, collagen and muscular fragments were present for all doses. Across both CH parameter sets, tissue fragments were only identified at higher doses(≥ 144s). In fully homogenized treatments, they were < 50μm.

### Quantitative assessment, areal analysis.

The percentage of tissue destroyed with each parameter set and dose is summarized in Fig. 2(b). BH destroyed a greater percentage of tissue within the measured ROI, at a lower dose, followed by the low-PRF and high-PRF CH parameter set. The results of a one sample t-test annotated in the same figure indicate the dose at which the average tissue destroyed (%) was statistically indistinguishable from 100% destroyed for each parameter set. For BH, this was at 30s with 94 ± 9% of the tissue destroyed. Approximately 85 ± 12% of the tissue was destroyed with the low-PRF CH at 144 s, and approximately 86 ± 12% was destroyed with the high-PRF CH at 288 s. The minimum average tissue destroyed (%) across all parameter sets and dose for a complete lesion was measured to be ~ 84%, which was set as the minimum threshold, as highlighted by the red line. All the treatments above this threshold were also correlated with the qualitative analysis findings to determine that the treatments were complete.

### Shear wave elastography analysis

#### Pre-treatment sample stiffness

**Figure 3:**
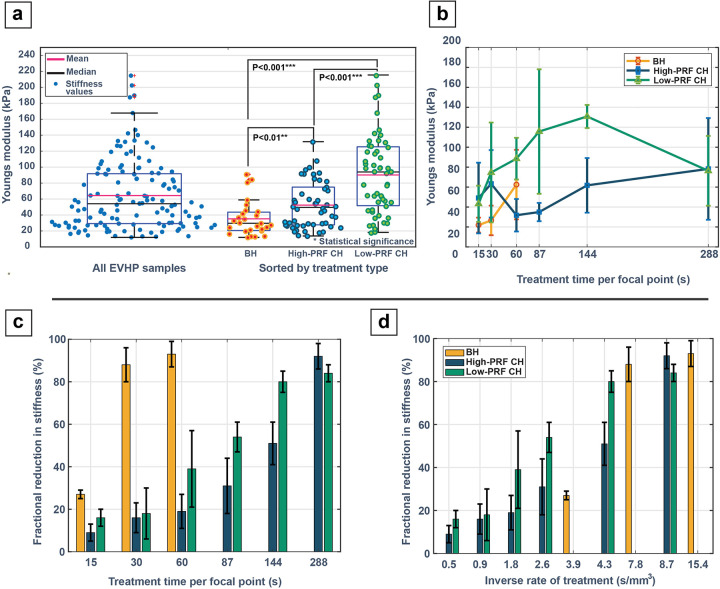
Shear wave elastography measurements **(a)** Pre-treatment young’smodulus (stiffness) of all the EVHP samples across all treatments segregated based on the treatment parameter set. *p-values* were derived from a 2-tailed 2-sample t-test. **(b)** The pre-treatmentstiffness of the slices that represent the center of the lesion segregated by dose and treatment parameters **(c)** Reduction in stiffness percentage post-treatment as a function of dosage (treatment time per grid point) and **(d)**Reduction in stiffness percentage post-treatment normalized by the individual transducer focal volume. The purple line indicates a SWE threshold that corresponds to a complete lesion homogenization.

The allocation of the treatment dose and modality in each sample was randomly assigned. However, the pre-treatment stiffness for the low-PRF CH treatments were significantly higher than the high-PRF CH and BH (P < 0.001), as indicated by a 2-tailed 2-sample t-test (Fig. 3 (a)).

Summarized in Fig. 3(b) is the pre-treatment stiffness sorted by dose for each parameter set. For BH, the mean pre-treatment stiffness was 37 ± 27.3(s.d.) kPa, with a median value of 26.16 kPa and an IQR = 22.3–41.6 kPa. For the high-PRF CH treatment group, the mean stiffness was 53.2 ± 31.6(s.d.) kPa, with a median value of 46.4 and an IQR of 28.6–72.4 kPa. For low-PRF CHs, the mean pre-treatment stiffness was 88.2 ± 43.3(s.d.) kPa, with a median value of 96.5 kPa and an IQR = 56 −118.6 kPa.

#### Post-treatment stiffness change analysis:

Post-treatment, SWE measured stiffness was reduced within the histotripsy treatment area with a clear contrast in the shear wave color map and a corresponding hypoechoic region observed on the B-mode for sufficiently long treatment times Fig. 3(c) shows the fractional reduction data across all the parameter sets at every dose. At the histologically identified complete lesion dosage points, towards the center of the lesion the change was − 88.4 ± 8.5(s.d.) % at 30 s for BH, while it was − 91.6 ± 6.0 (s.d.) %. for the high-PRF CH at 288 s and − 79.9 ± 5.4 (s.d.) % for the low-PRF CH at 144 s.

Due to the different focal volumes of the CH and BH transducers respectively, it is difficult to directly compare volumetric treatment rates for these data. Nonetheless, we approximately estimated the rates based on the volumes treated and time required to achieve a complete ablation for the entire lesion. The inverse rate of treatment (time per unit volume for the specific treatments) plotted as a function of the fractional reduction in the stiffness percentage, is shown in Fig. 3(d), where the stiffness data was normalized by the individual transducer focal volume to estimate the rate for these specific treatments. This translated to a rate of 13.9 mm^3^/min for low-PRF treatments followed by BH at 7.7mm^3^/min and high-PRF treatments at 6.9mm^3^/min.

#### Regression analysis between histological analysis and shear wave data

**Figure 4:**
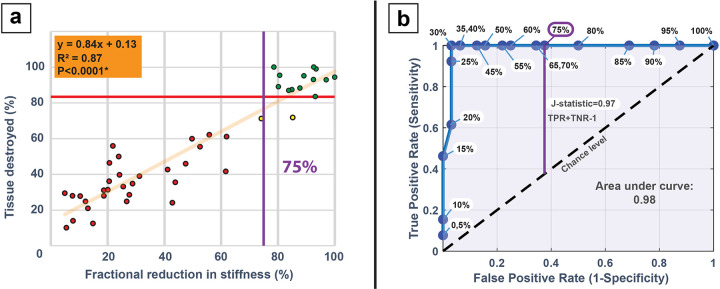
Regression analysis of shear wave and histology measurements: **(a)** Linear regression analysis between the shear wave reduction stiffness and homogenized tissue percentage quantified using the HLQE algorithm. The red line indicates the 84% threshold calculated from histological assessment from prior section which is the minimum average tissue destroyed (%) across all parameter sets and dose for a complete lesion. The red cluster represents completely fractionated lesions, with yellow, red clusters representing partial lesions. **(b)** An ROC curve was constructed for every shear wave threshold, with the Youden’s j statistic predicting a 75% reduction in stiffness pre-post as measured by shear wave to be a good indicator for histologically observed complete lesions.

Regression analysis was performed to compare the destroyed tissue percentage vs. the stiffness reduction via SWE, and the results are shown in Fig. 4(a), with R^2^ = 0.87 and a t-test of the slope coefficient where P < 0.0001*, indicating a significant relationship between the two parameters. The individual scatter points are also indicative of three clusters, with green cluster representing completely fractionated lesions and the red, yellow clusters representing progressing partial lesions.

The ROC curve generated to determine the relative predictive strength of the SWE measurements for the assessment of tissue disintegration is shown in Fig. 4(b). The area under the ROC curve (AUC) was 0.98, which indicates the high performance of the classification criteria based on the SWE threshold in distinguishing full lesions from partial lesions. The Youden’s J statistic calculated as 0.97 further captures the performance of the dichotomous test, where a higher value is considered better. From the J statistic, the optimal threshold for stiffness reduction was identified as 75% from a premeasured value. These two observations emphasize that shear wave-based feedback can be a reliable indicator of a histologically observed lesion.

## Discussion

Previous histotripsy studies of BPH examined parameters and dose in a dog model^22^, but the canine prostate tends to be predominantly glandular; therefore, the identified treatment may be an underestimate of the actual dose needed in humans. This may also explain some of the inefficacy of the results in a pilot clinical trial^32^ using histotripsy to treat BPH. As such, this work is a step toward understanding how to optimize parameters to ablate fibrotic or fibromuscular tissue more rapidly and to evaluate treatment monitoring techniques for predicting successful treatment completion. This study compared the dose effects of different parameters of cavitation cloud histotripsy and boiling histotripsy in mechanically ablating *ex vivo* human benign prostatic hyperplasia tissue. Although a substantial body of literature exists on testing histotripsy modalities with different parameters, few studies have compared the ablation efficacy of different modalities for a single tissue type. Additionally, *in vitro* and *in vivo* data indicate the differential susceptibility of different tissues to a given set of parameters. For example, Vlaisavljevich et al demonstrated that mechanically tough tissues require a greater dose to completely erode than soft tissues^33,34^, which was also observed in cholangiocarcinoma^29^ and uterine fibroid^30^ treatments. BPH tissue is a heterogeneous mixture of soft glandular tissue and tough fibromuscular tissue, the composition of which can vary widely between patients.

All three parameter sets produced complete ablation at sufficient doses. On a point-by-point basis, the BH parameters tested here produced liquefaction more rapidly. This may be related to the high duty cycle of this treatment (1%) compared to that of the other parameters (0.2% for high-PRF CH and 0.03% for low-PRF CH). However, the difference in focal volumes between the BH and CH transducers and scanning parameters limit our ability to conclude whether these are more effective volumetrically. Through an analysis where we estimated the volumetric treatment rates, low-PRF CH parameters produced a greater rate than both BH and high-PRF CH. The volumetric rate for BH estimated in this study is consistent with a recently published study involving treating the ex-vivo human prostate with similar BH parameters^30^. One potential explanation for these differences, as observed in previous studies^24,35^ is that high-PRF treatments suffer from cavitation memory effects. The bubbles generated from the previous pulse persist for longer durations in tougher media ^24^ before completely dissolving. If a second pulse arrives prior to the previous bubble dissolution, it cavitates bubbles in the same location. This can lead to sparse cloud formation with only isolated small regions of damage. Low-PRF CH, on the other hand, creates denser clouds that nucleate bubbles in different locations with each pulse, due to sufficient time for the bubbles to dissolve, and therefore cover the volume of the tissue to form a more complete homogenate lesion faster. A potential reason for estimating the volumetric rate as described in this paper versus estimating from histological slides, was due to artefacts in tissue processing for staining including tissue shrinkage that occurs during the fixation process. Another challenge with comparison is that despite randomization of the treatments, the initial stiffness of the tissue on average for the low-PRF treatments was considerably higher than that of the BH parameters or high-PRF CH parameters. Also, BH treatments had a slightly higher overlap, about ~ 70% in comparison to CH treatments at ~ 50%, meaning a slightly higher dose per unit area; therefore, the actual efficacy of low-PRF in comparison to BH could be underreported. Despite these challenges, it is clear simply from the comparison of the two sets of CH parameters that the efficacy of treatment for fibrous BPH tissue can be significantly improved with the use of parameters different than those attributed to early BPH studies, indicating a need for optimization of parameters for this application prior to clinical trials.

Thermal effects were seen with both BH and high-PRF treatments. This was validated by the presence of globular collagen that is consistent with findings from Wang et al ^15^, when using higher duty cycle BH pulses. At the lesion borders, there was presence of frayed collagen, similar to BH outcomes of treated *ex vivo* prostate cancer samples ^31^, and we also observed some thermal damage in the form of blanched tissue at the outer rim of the lesion which was reported in other studies using 10ms pulses^15,36^ BH parameters. There was also an increased presence of tissue fragments possibly due to the vapor cavity created by the boiling bubble leading to an acoustic fountain effect that causes tissue disruption into several smaller fragments^37^. Broken collagen and muscle fibers make up the composition of these fragments, indicating origins from predominantly fibromuscular treatments. In treatments primarily positioned in glandular areas, tissue fragments were rarely observed, as they tend to completely homogenize. The CH parameters displayed reduced evidence of thermal effects, but the presence of more globular collagen in the high-PRF treatment group was potentially due to the increased duty cycle used in this study and the greater dose for liquefaction.

Shear wave elastography was found to be well correlated with lesion progression histologically and may be a valuable tool to determine treatment completion in the clinical setting. SWE estimates the Young’s moduli of the prostate samples with high accuracy^38^, and the values reported here are consistent with those reported from transrectal SWE in patients^39–41^. From the *ex vivo* studies, we encountered samples with different initial stiffnesses, and a similar variance is expected in a clinical scenario. Along with attenuation and aberration affecting the acoustic pressures achieved *in situ* at the focus, the homogenization rates will differ for each treatment. It is therefore essential to obtain feedback to assess the progress of treatment to provide an opportunity to adjust the dose for each location, to optimize treatment. B-mode provides only qualitative feedback, and it can be difficult to track the hypoechoic treated region due to the presence of hyperechoic bubbles that can persist for longer time intervals. Recent studies have employed Doppler-based measurements^42,43^ that track the speed and direction of cavitation bubbles as an indicator of homogenization, and may also offer quantitative information. Accounting for different starting conditions, we found a threshold of > 75% reduction from the initial stiffness to indicate complete histologic disintegration. This feedback can allow optimization of treatment times specific to each case. One of the inherent limitations of shear wave measurements in an *ex vivo* setting is that saturation effects arising due to solid tissue and liquid boundary artifacts tends to overestimate the stiffness if the size of the sample is too small, thus we were careful to avoid treating near such boundaries.

In this study, we also successfully showed the utility of an entropy-based analysis for the quantification of lesions in Masson’s trichrome images to better assess the treatment effects for future treatment planning. Currently, there does not exist an automated segmentation methodology for differentiating homogenate lesions from tissue based on texture, and the HLQE algorithm can also be extended for the analysis of other stains to look at damage patterns.

One limitation of this study is the variation between size and shape of samples that necessitated treatment of different volumes of tissue. However, the dose per focal volume was constant between samples, and this variance is not expected to affect the measurements made since the minimum grid size was still large enough to perform shear wave elastography and histologic analysis. Future studies will aim to use a more uniform grid size to quantify the true treatment area vs the intended area. We aim to carry out a more direct comparison across several histotripsy parameters and strategies^44 45^ using hardware that can perform both BH and CH potentially through a transrectal approach^46^ that can circumvent the challenges associated with delivering ultrasound through a transperineal/transabdominal approach.

To summarize, we compared homogenization of tough fibrotic human *ex vivo* benign prostatic hyperplasia tissue via three different sets of histotripsy treatment parameters at varying doses. The results demonstrated that alternative treatment parameters can achieve more rapid ablation of BPH tissue compared to those attributed to previous BPH studies. This work also established the utility of shear wave elastography as a reliable tool for obtaining feedback during treatment to determine the endpoint. A threshold reduction in SWE-measured stiffness for of approximately ≥75% was found to correlate well with complete lesion formation. These results and feedback offer strategies to achieve more efficient treatment of BPH tissue by histotripsy in future trials.

## Methods

### HIFU equipment

The experimental setup consisted of two transducer setups for administering CH and BH along with their respective image guidance platforms.

- **Cavitation Histotripsy**: CH was administered using a 700 kHz 18-element transducer with an *f-number* = 0.85, a focal distance of 11 cm and an aperture of 13 cm. The transducer was constructed from piezoelectric elements (880 ceramic, APC International Limited, Mackeyville, PA, USA) and fixed using a tungsten-epoxy matching layer to a 3D printed housing lens made from Accura 60 photopolymer (Protolabs, Plymouth, MN, USA). The transducer has a central cavity to coaxially host an M5Sc ultrasound imaging probe from GE Healthcare (Chicago, IL, USA) on a Vivid E95 platform to provide ultrasound B-mode image guidance. A custom-built class D amplifier^47^ controlled by a PC was used to electrically power the transducer through a matching network.
- **Boiling Histotripsy**: BH was administered using a 1.5 MHz 12 element transducer with an *f-number* of 0.77, a focal distance of 5.6 cm and an aperture of 7.3 cm derived from this study ^48^. The transducer was built using flat trapezoidal elements and bonded to a rapid prototyped focusing lens with a matching layer. B-mode image guidance was provided by a coaxially hosted P4–2 probe on a Sonix RP Ultrasonix legacy system (BK Medical, now part of GE Healthcare, Chicago, IL, USA). The same amplifier system as in the previous section with an added capacitor bank to sustain the longer BH pulses was used to power the transducers through a matching network.

### Transducer characterization

Both transducers were characterized for their focal pressure waveforms using a fiber optic hydrophone (FOPH2000, RP Acoustics, Stuttgart, Germany) at a low duty cycle. The − 6 dB beamwidth along the three axes was measured using a lipstick hydrophone (HGL-0085, ONDA Corp, Sunnyvale, CA, USA) at low pressure under linear propagation conditions. For the CH transducer, the − 6 dB beamwidth measured 13.1 × 2.2 × 2.2 mm (axial × lateral), which translates to a 0.265 cm^3^ ellipsoidal focal volume. For the BH transducer, the beam measured 6.7 × 1.0 × 1.1 mm, which translates to a 0.034 cm^3^ focal volume.

### Pulse parameters and dose

The following pulse parameters (treatment modalities) were used to treat the BPH samples. CH was administered with two pulsing schemes: (i) a high pulse repetition frequency (PRF)-shorter pulse duration treatment (used previously with a clinical system ^22^,^23^) and (ii) a low PRF, longer-pulse duration scheme and (iii) with grids ranging from a minimum of 9 foci points up to a maximum of 25 points with an overlap of 1.15 mm (Table I). BH was administered at a PRF of 1 Hz and 15000 cycles in a similar grid fashion with an overlap of 0.75 mm. The treatments were administered in a raster scan moving the focus in two dimensions orthogonal to the acoustic axis. The pulse parameter sets are summarized in Table 1.

Based on a prior CH-based clinical study ^32^, it was determined that the average treatment volume was 10.8 cm^3^. From the average total treatment time and focal dimensions of the clinical source, we estimated that the average time per focal volume to treat was approximately 30 seconds, and this time was used to determine the duration of treatment for this study. The duration of CH treatment at each point was set to 15, 30, 60, 87, 144 or 288 seconds. With overlapping treatment points spaced at one-half beamwidth, this would translate to a maximum of ~ 10k pulses for low-PRF CH and ~ 500k pulses for the high-PRF CH at the highest time dose (288 s) at the center of the lesion grid. The highest time of 288 seconds was also based on optimization experiments performed in a tissue phantom we developed with mechanical parameters similar to those of fibrous tissue^24^. BH was only performed for time points ≤ 60 seconds based on prior literature, which suggested that fully homogenized tissue can be achieved in softer nonfibrotic models at 15–50 seconds^14^.

In both cases, treatment was delivered at focal pressure amplitudes 20% above the threshold at which sustained cavitation or boiling was first observed on B mode ultrasound in each tissue sample while incrementally increasing the output.

### Experimental approach

#### Tissue preparation

De-identified human prostate tissue samples (n = 23) were obtained from patients undergoing simple prostatectomy procedures for BPH at the University of Washington (UW) Medical Center, Seattle, WA. Patients provided informed consent through NWBioTrust, Seattle, WA which also handled the sample collection and transfer according to an UW Human Subjects Division (HSD) institutional review board (IRB) approved protocol. The study was determined to be an IRB exempt non-human subject research by UW HSD IRB and all experiments were performed in accordance with institutional and National Institutes of Health (NIH) guidelines.

Samples ranged from 5–10 cc and were acquired within an hour of the operation. The samples were stored in phosphate-buffered saline (PBS) until they were embedded in 1.5% UltraPure^™^ Agarose (Thermo Fisher Scientific, Waltham, MA, USA) for sonication experiments, which were performed within 24 hours of extraction. The 1.5% agarose solution was made by dissolving 3 g of agarose in 230 mL of deionized water and boiling for 6 minutes, yielding ~ 200 mL of the final mixture. The solution was then transferred into a casting container and allowed to cool to approximately 40°C before the sample was embedded.

#### Stiffness measurement, sonication setup, treatment and preparation of samples for histology

**Figure 5:**
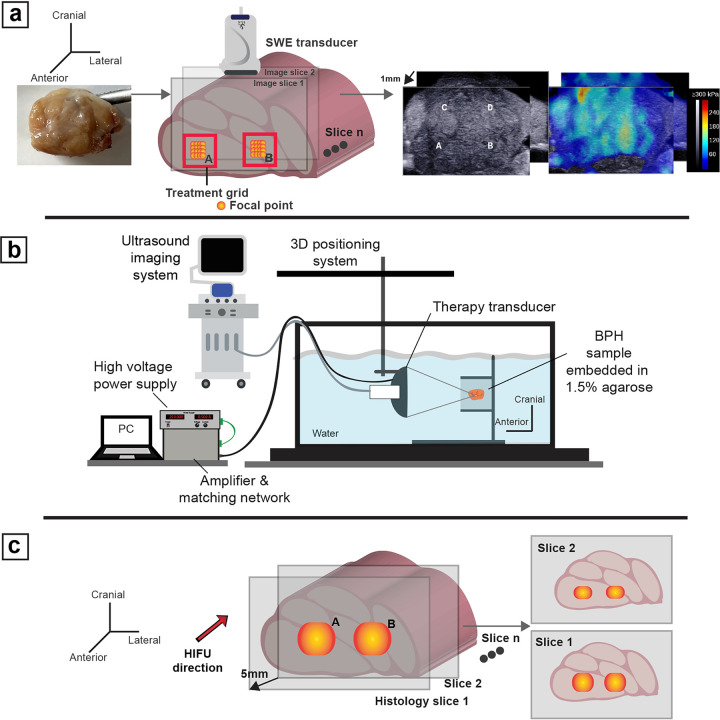
Illustrations of the experimental setup **(a)** Shown from L-R the assumed orientation of the sample with respect to the transducer. The SWE setup captured stiffness measurements at 1 mm intervals along the entire sample transversing from anterior to posterior. An example B-Mode and SWE image of the sample measured using the apparatus is shown. **(b)** Experimental setup for *ex vivo*treatment of human prostate tissue. The setup shows the samples embedded in 1.5% agarose and placed in a degassed deionized water chamber. The therapy transducer with a coaxiallyarranged imager is then submerged into the tank with the focus of the therapy positioned within the sample. The therapy transducer parameters are controlled externally using a custom FPGA board and powered by a class D amplifier. Multiple therapy points are delivered as shown by orange dots in a raster fashion. **(c)** After treatment, the fixed tissue was sectioned for histological analysis in a manner encompassing all the treatment grids.

The baseline stiffness of the samples prior to embedding in agarose was measured by SWE using a 256-element linear SL 15 – 4 transducer from Supersonic Imagine (AiXplorer, Aix-en-Provence, France) mounted on a 3-axis positioner^24^. The sample was placed in a fixture aligned with sound-absorbing rubber pads to reduce reverberation and filled with saline solution. The acquisition settings involved using the ‘general’ preset, set to penetration mode. The regions for treatment were identified by initial coarse measurements using the on-board measurement tool. Next, the SWE maps were collected in 1-mm increments capturing most of the tissue along the length of the sample to generate a 3-dimensional SWE stiffness map. The SWE image plane was perpendicular to the plane in which the therapy was delivered to capture all the treatments in a single plane. Figure 5(a) illustrates the orientation, an example of the B-mode image, and the corresponding SWE image.

The agarose-embedded sample was placed in a fixture that provided minimal disruption to the acoustic pressure field and lowered into a tank filled with deionized and degassed water, ideally at approximately 20–25% O_2_ saturation, to mimic conditions within the human body. The transducer was attached by optical rods to a motorized positioner and controlled via MATLAB (The Mathworks, Natick, MA, USA). The setup is shown in Fig. 5(b). Treatment grid points were positioned with 50–70% overlap and delivered in a raster scan with the focus moved from the anterior to the posterior of the sample. Treatment was delivered at least 5 mm from the anterior surface at all points, such that the entire treatment was confined within the sample based on prior studies^49^. The threshold pressure was determined in a volume distinct from the treatment grid, where the amplifier voltage was increased until a cloud was observed, which was verified using B-mode imaging. Treatment was then delivered at + 20% above threshold to account for threshold changes from tissue inhomogeneities. A minimum of a 5-mm gap between each treatment grid was maintained to ensure no overlap between different treatment regimes. The number of treatments and the grid size were determined based on the size of the sample available. Post-treatment, all the samples were removed from the agarose gel, and stiffness measurements were collected by SWE in a similar fashion as outlined above. All the SWE data were then exported for offline processing.

Next, the samples were fixed in a 10% neutral buffered formalin solution for at least 72 hours. Each sample was then segmented into multiple blocks of 5 mm thickness covering all treatment grids as shown in Fig. 5(c), dehydrated in 70% ethanol, and sent offsite (Scientific solutions, Fridley, MN) for histological processing. Whole mount sections (5 um) taken from each block were stained with Masson’s trichrome (MT).

### Histological evaluation

#### Qualitative analysis

Histological analysis served as the primary method for evaluating treatment effectiveness. Whole mount MT-stained sections were scanned using an Aperio slide scanner (Leica Biosystems, Illinois, United States). Based on our extensive experience analyzing histotripsy lesions qualitatively, we classified the lesions from this study into the following categories to assess treatment outcomes:

- **Partial lesion**: Damaged pockets coinciding with bubble clouds, as observed in high-PRF bubble cloud images^35^. Homogenized areas that start to appear with larger tissue fragments (broken collagen strands, muscular fibers).
- **Complete lesion**: Fully homogenized lesion with minimal tissue fragments ( < ~ 50μm) and cell debris.

### Quantitative assessment, areal analysis

In this study, we used textural analysis of histological images to quantify lesion area as a function of dosage for each treatment parameter set using a custom algorithm called Histotripsy lesion quantification by the entropy analysis algorithm (HLQE). For every parameter set and dose, across n = 3 samples, one histological slice representing the “center of the lesion” was chosen for quantification to measure the maximum treatment effect. Characteristically, histotripsy homogenizes tissues and reduces their structure to a uniform field of debris. As such, an appropriate method to identify homogenized tissue vs. intact tissue is based on the variation in structure within the image, which is done here through measurement of image entropy. To estimate the fraction of intact tissue within a lesion, a local entropy filter was used on an image of each histological section. Within a subregion of 25×25 pixels in the image, the entropy filter calculates the associated entropy S,

1 $$S=-\sum \;p{log}_{2}p$$

where p is the normalized histogram of pixel intensity counts. Entropy as a function of position is displayed, with areas of low entropy indicating disrupted tissue due to their uniformity and lack of tissue structure. These images were thresholded and converted to binary images using Otsu’s method (graythresh function in MATLAB), and areas below the threshold were considered part of the disrupted tissue. Within the region of interest (ROI) consisting of the intended treatment volume, a total of 48 random areas of r = 20 pixels were sampled, and the fraction of area disintegrated was calculated as a quantitative estimate of the completeness of treatment. A detailed description of the HLQE algorithm processing steps are included in the attached supplemental along with performance evaluation results compared against manually segmented histology slides.

A one-sample t-test was performed for the average percentage of tissue destroyed from the histological slides using the HLQE algorithm across all treatment modalities. The null hypothesis is that the measured mean percentage of tissue destroyed in the sample is equal to 100%, indicating a complete lesion, $H_{0}:\beta 1=100\%$ with the alternate hypothesis $H_{1}:\beta 1\neq 100\%$ with a = 0.05. The outcome of this test will determine the doses at which the average percentage of tissue destroyed is indistinguishable from 100% destroyed tissue. Also, we will establish the threshold value (minimum average percentage of tissue destroyed) to be used for the receiver operator characteristic analysis to be discussed later below.

### Shear wave elastography analysis

To account for the different starting stiffnesses between the samples, the pre- and post-treatment stiffness changes were measured as the fractional reduction in stiffness from the initial value in the respective grid. The stiffness measurements were processed offline using a custom-written MATLAB algorithm that parametrized the SWE scale from the exported JPEG image of a slice and created an RGB map corresponding to the displayed stiffness for all the pixels present in the on-screen scale. From both pre- and post-treatment SWE maps, the measurement representing the center of the lesion was chosen. Within these planes, the center of the region of interest was manually selected using a freeform ellipse tool of radius (2–4 mm), and the average stiffness within that region was calculated to avoid boundary artifacts often encountered when measuring near the edge of the treatment grid. This process was repeated for all n = 3 samples for each treatment parameter set and dose. From the change in stiffness and treatment time, the stiffness change as a function of the rate of reduction (time spent per individual focus) was calculated and plotted. To normalize the different beamwidth volumes of the foci used between the two different CH and BH transducers, the fractional reduction in stiffness was plotted as a function of the inverse rate of treatment, i.e., the dose normalized by the focal volume.

### Regression analysis between histological analysis and shear wave elastography

An analysis was performed to determine whether there were relationships between the histological intactness of the tissue and the SWE data:

- Student’s t-test for the slope coefficient was performed to determine whether there was a significant correlation between changes in stiffness and tissue disintegration. The null hypothesis is that the coefficient $H_{0}:\beta 1=0$ indicating no significant relationship between the regression analysis variables, with the alternate hypothesis $H_{1}:\beta 1\neq 0$ with a=0.05.
- A receiver operating characteristic curve (ROC) was constructed to determine the utility of SWE as a predictor of complete lesion formation. Lesions with average tissue destroyed (%) greater than the threshold value determined from the histological quantitative analysis and correlated with the qualitative analysis were deemed a fully homogenized lesion. The area under the curve (AUC) was calculated to determine the predictive value of SWE. The optimal threshold/cutoff was determined by using Youden’s J statistic, which captures the performance of the classifier. It is given by the following formula.

2 Jstatistic=TPR+TNR−1

and has a value between 0–1. The index was calculated for all the threshold points on the ROC curve, and the maximum value of the index was used to determine the cutoff point.

## Figures and Tables

**Table 1:** Pulse parameters used in the study.

Treatment Modality	Parameter Set Name	Pulse Repetition Frequency (PRF) (Hz)	Pulse Duration (Cycles)	Operating Frequency (MHz)	Duty Cycle (%)	Time Duration per point(s)	Peak Pressure at focus (MPa)
**Cavitation Cloud Histotripsy (CH)**	High-PRF	500	3	0.7	0.21	15–288	P_+_54–108 P_−_ 14–20
	Low-PRF	10	20		0.03	15–288	P_+_95–108 P_−_ 16.6–20
**Boiling Histotripsy (BH)**	N/A	1	15000	1.5	1	15–60	P_+_68–86 P_−_ 14.5–15.8

## Data Availability

The datasets generated during and/or analyzed during the current study are available from the corresponding author on reasonable request.
